# Analyses of Individual Singly Charged Ions Using a High-Field Orbitrap Analyzer

**DOI:** 10.1021/acs.analchem.6c02125

**Published:** 2026-05-12

**Authors:** Elena Giaretta, Evolène Deslignière, Eduard H. T. M. Ebberink, Tobias P. Wörner, Arjan Barendregt, Kyle L. Fort, Alexander A. Makarov, Albert J. R. Heck

**Affiliations:** † Biomolecular Mass Spectrometry and Proteomics, Bijvoet Center for Biomolecular Research and Utrecht Institute for Pharmaceutical Sciences, Utrecht University, Utrecht 3584 CH, The Netherlands; ‡ Université Paris-Saclay, CNRS, Institut de Chimie des Substances Naturelles, UPR 2301, 91198 Gif-sur-Yvette, France; § Thermo Fisher Scientific GmbH, Bremen 28199, Germany

## Abstract

Orbitrap-based charge detection mass spectrometry (CDMS) enables the mass analysis of biomolecules at the individual-ion level. Recording longer transients boosts resolution and sensitivity for CDMS measurements. However, singly charged analytes (∼1–2 kDa) remained elusive to Orbitrap-based CDMS due to their low signal-to-noise ratios (S/N). To overcome this limitation, we implemented CDMS on a high-field Orbitrap mass analyzer (HF-OT, Exploris 480 mass spectrometer) coupled to an external data acquisition system. The HF-OT analyzer should theoretically improve the resolution by 1.7-fold and the S/N by 1.4-fold compared to a standard Orbitrap analyzer (S-OT, UHMR mass spectrometer). We first adapted an Exploris 480 mass spectrometer to allow robust detection across an extended *m*/*z* range (∼1 kDa to 500 kDa) and enable transient recording up to 20 s. Experimentally, the HF-OT outperformed the S-OT in charge and mass accuracy for a variety of systems, such as insulin, BSA, and a monoclonal antibody, and allowed us to achieve an unprecedented mass resolution far above 3 million even at *m*/*z* ∼ 4250. On the lower side of the *m*/*z* range, the modified HF-OT allowed the recording of individual molecules carrying just a single charge. For the first time, the peptides angiotensin I and bradykinin could be unambiguously detected and mass analyzed above the noise threshold (S/N = 2), albeit only at extended transient times, creating a new lower mass and charge limit for Orbitrap-based CDMS.

## Graphical abstract

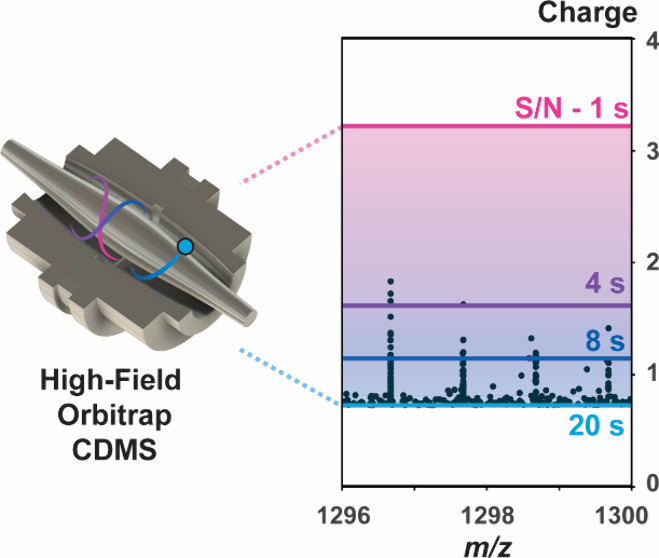

## Introduction

Charge detection mass spectrometry (CDMS) enables the characterization of polydisperse and high-mass assemblies, relying on the detection of individual particles to circumvent drawbacks of ensemble-based mass spectrometry (MS).^1,2^ In standard MS experiments, molecular masses are not measured directly but are derived from the relative mass-to-charge (*m*/*z*) ratios of millions of ions, requiring that charge states are resolved.^3^ However, for heterogeneous and large biomolecules, charge state distributions often overlap, obstructing accurate charge determination. CDMS simultaneously measures the *m*/*z* ratio and the charge (inferred from the amplitude of the image current) of each individual ion, circumventing the need for charge deconvolution.^4^

Charge determination of individual particles was initially exclusively performed on modified, home-built mass instruments (time-of-flight, electrostatic linear ion trap, ELIT).^5–7^ ELIT-based CDMS has since undergone major developments, achieving 0.2e single-ion charge precision and almost perfect charge-state assignment in advanced home-built instruments, and is now available as a dedicated commercial platform such as the Xevo CDMS, which combines an optimized ELIT with a low-noise charge-sensitive amplifier to enable fast, high-precision single-ion charge measurements.^8^ On another front, the CDMS with the optional Direct Mass Technology (DMT) mode was implemented on commercially available Orbitrap instruments,^9,10^ among which the EMR and UHMR mass spectrometers had already been widely adopted for high-resolution native MS.^11^ A particularly attractive feature of Orbitrap-based CDMS is its ability to be performed on any Orbitrap mass analyzer when transient data are accessible through adaptation in the software and/or an external data acquisition system. The UHMR platform, enabling an extended *m*/*z* range, has so far been the go-to instrument to perform Orbitrap-based CDMS. Recent applications of Orbitrap-based CDMS include the mass analysis of vaccines and gene-delivery vectors, membrane proteins, ribosomes, mRNA, and DNA plasmids.^12–18^ Heavily glycosylated proteins, such as IgM antibodies or the Covid-19 Spike protein trimer, can also be readily mass analyzed by CDMS, where standard native MS falls short as ion signals are no longer charge state resolved. All this makes CDMS particularly interesting for the characterization of biopharmaceutical products that continuously are expanding in molecular complexity, involving for instance also glycosylation-related heterogeneity.^19–21^

Since its introduction in 2020, Orbitrap-based CDMS has undergone significant advancements to push boundaries in sensitivity and mass resolving power, supported by more sophisticated data processing systems and algorithms.^22–27^ The implementation of ultralong transients (∼25 s) on the UHMR mass spectrometer has enabled higher mass resolution and signal-to-noise (S/N) ratios, and consequently enhanced charge accuracy.^28^ While this method was shown to be particularly well-suited for the detection of high mass assemblies, an improved S/N also could open new possibilities to analyze smaller proteins or even peptides. Measuring individual ions of low *m*/*z* species with few charges remains constrained by (i) the stability of ion trajectories in the Orbitrap analyzer upon collisions with the background gas,^29–31^ and (ii) the maximum transient duration that can be recorded hampering individual ion signals to get above the noise level.

We recently partly addressed the ion stability issue by employing an electron-capture charge reduction (ECCR) strategy.^32,33^ By shifting individual ions to higher *m*/*z* regions, ECCR reduces their orbiting path length and kinetic energy, resulting in fewer and less energetic collisions with gas molecules. ECCR-CDMS significantly improved the ion survival at extended transients, even allowing to detect doubly charged cytochrome (cytoC) ions, using transients of 24 s. This approach brought us closer to recording singly charged individual ions, but this fundamental goal remained out of reach. From the data and theory, we could predict that a perfectly stable 1+ ion would require a transient of at least ∼42 s to lead to a signal above the noise level (S/N = 3) for a standard field Orbitrap mass analyzer (S-OT, D30).^28^ Unfortunately, hardware constraints (i.e., electronic instabilities) currently limit the maximum transient duration to 25 s which precludes the measurement of singly charged ions with CDMS with this Orbitrap mass analyzer configuration.

One less-explored avenue for CDMS analysis is the use of high-field Orbitrap (HF-OT, D20) mass analyzers.^34^ The HF-OT is 1.5 times smaller than the S-OT mass analyzer, and its central electrode is relatively thicker while utilizing a high voltage of 4 kV. The reduced spacing between central and outer electrodes increases the effective field in the trap, which causes ions to oscillate at higher frequencies. This higher oscillation frequency leads to an increase in *m*/*z* resolution per unit time, with a theoretical 1.7-fold improvement over the S-OT. In parallel, the smaller geometry and reduced capacitance of the system improve the S/N ratio of the image current, yielding a 1.4-fold enhancement. As the noise of the image current is the main source of charge uncertainty and S/N scales with charge resolution, this improvement also directly enhances charge accuracy. These combined effects are expected to benefit not only high-mass assemblies but also lower mass ions carrying only a few charges, such as peptides.

In the present work, we explored the potential of the widely used Orbitrap Exploris 480 mass spectrometer for individual-ion detection, and we evaluated the performance metrics achieved on a HF-OT analyzer compared to the S-OT analyzer for various analytes across a wide range of masses.^35^ Using as proof-of-concept samples bovine serum albumin (BSA) and insulin, we first experimentally demonstrated the predicted improvement in both resolution and S/N, leading to enhanced charge accuracy. We also developed a robust data processing workflow tailored for ions with low charges to avoid mistakenly processing noise peaks and to reliably discard ions drifting in their frequency domain. Thanks to the high sensitivity of the HF-OT analyzer and the new processing approach, we demonstrate the detection and mass analysis of singly charged individual ions of two model peptides (i.e., bradykinin and angiotensin I), reaching, for the first time, measurement of 1+ ions with Orbitrap-based CDMS. Additionally, we aimed at recording individual ions for high mass macromolecular assemblies, expanding the *m*/*z* range of HF-OT CDMS applications, and achieved an unprecedented mass resolution at *m*/*z* ∼ 4250 of above 3 million. We were able to transmit and detect individual ions of an intact antibody (∼150 kDa) and were also successfully resolved oligomers of apoferritin (apoF, ∼500 kDa). Overall, the acquired data reveal that the HF-OT is a very versatile mass analyzer which outperforms the S-OT across a wide mass range in CDMS analyses.

## Materials and Methods

### Sample Preparation

Bradykinin acetate, angiotensin I human acetate salt hydrate, insulin from bovine pancreas, cytoC from equine heart, BSA, and trastuzumab were purchased from Sigma-Aldrich (Darmstadt, Germany). Heavy chain only apoF in recombinant form was acquired from Thermo Fisher Scientific (Waltham, United States), marketed as Vitro-Ease apoferritin. Insulin, trastuzumab, and apoF were subjected to buffer exchange into 150 mM aqueous ammonium acetate at pH 6.9 using Amicon (Merck KGaA, Darmstadt, Germany) centrifugal units with, respectively, 3, 50, and 100 kDa molecular-weight-cutoff (MWCO). BSA and cytoC were buffer exchanged to the same conditions with Micro Biospin 6 MWCO centrifugal unit from Bio-Rad (Bio-Rad Laboratories, Hercules, United States). To meet the concentration requirement for individual ion-measurements, samples were further diluted in ammonium acetate prior to analysis. Each sample was loaded in approximately 2 μL aliquots into in-house pulled, gold-coated borosilicate capillaries for nanoESI.

### Instrumentation for Ultralong Transient HF-OT CDMS

All CDMS experiments were conducted on a modified Orbitrap Exploris 480 mass spectrometer (Thermo Fisher Scientific, Bremen, Germany). An external high-performance DAQ system, the FTMS Booster X2 (Spectroswiss, Lausanne, Switzerland), was coupled to the instrument to enable a 20-s acquisition, as described elsewhere.^28,46^ Hardware constraints currently limit the maximum transient duration to 20 s and no further hardware modifications were made. However, software modifications were implemented to enable ion trapping and the acquisition of ultralong transients over an extended *m*/*z* range. In the research-grade instrument control software, parameters for acquiring ultralong transients and accessing advanced tuning settings were adjusted according to the specific analyte (Table S1).

### Acquisition Parameters

The source capillary temperature was maintained at 250 °C, and the capillary voltage was set between 1.2 and 1.4 kV. In-source fragmentation, HCD voltage, injection time, time-domain sampling frequency were tuned for each analyte (for detailed parameters, see Table S1). Nitrogen was utilized as collision gas, with as exemplary value an ultrahigh vacuum (UHV) readout of 1.83 × 10^–11^ mbar for BSA. Data were collected for 60–180 min using acquisition strategies described elsewhere.^28^

### Transient Processing

Raw signals from the FTMS Booster X2 recordings were initially converted into.h5 files using Peak-by-Peak version 2024.05.0 (Spectroswiss SARL, Lausanne, Switzerland). The resulting extended time-domain transient data were processed using an in-house developed Jupyter Notebook with associated Python functions library. ApoF and mAb data processing follows the Supplementary code provided elsewhere following the ‘frequency chasing’ approach and standard deviation filtering (threshold 1 and 0.25 for *m*/*z* and intensity).^28^ Duplicate ion traces are removed with the rolling approach (standard deviation cutoff 1.0 and 0.1, respectively, Figure S1). An internal calibration procedure was applied to compensate for intensity drift.

For analytes below 150 kDa, a novel data processing workflow was developed. In brief, cumulative time-domain transients (0–1 s, 0–2 s, ..., 0–20 s) were zero-filled three times and apodized using a Hamming window (except for mFT in Figure 1b, c and Figure S2a, b no apodization and for aFT in Figure S2a, b half-Kaiser apodization, β = 0). The processed segments were Fourier transformed from the time domain to the frequency domain using the Fastest Fourier Transform in the West (FFTW) algorithm,^47^ and the resulting spectra were computed in magnitude mode. Signal inspection revealed that, for transient lengths exceeding 20 s, all individual peaks gradually shift to lower frequencies. This behavior limits the maximum transient duration on our Orbitrap Exploris 480 mass spectrometer.

**Figure 1:**
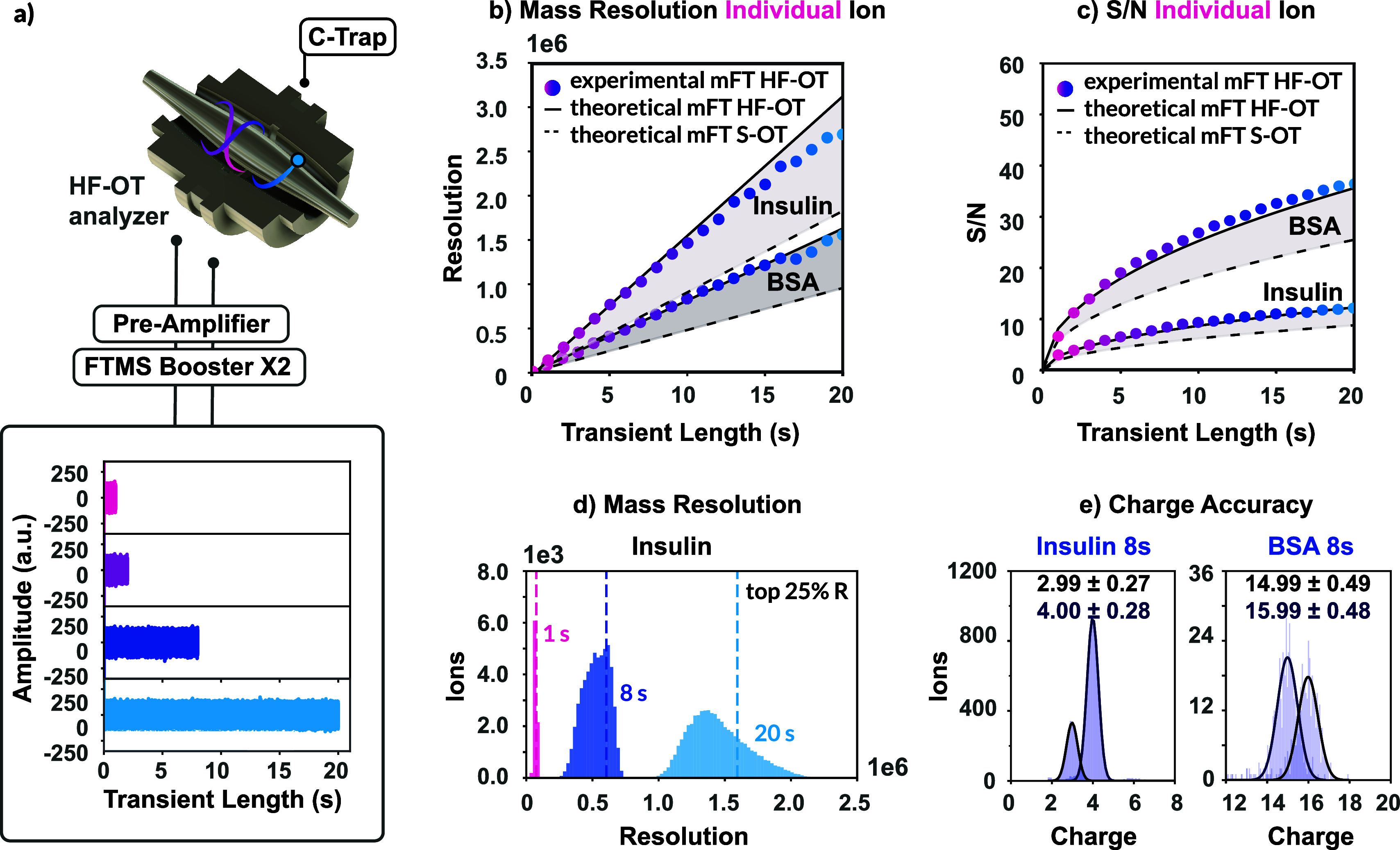
Demonstration of enhanced resolution and S/N on the HF-OT analyzer for individual-ion CDMS. (a) Depiction of the HF-OT analyzer and the instrument coupling to an external high-performance DAQ system FTMS Booster X2 (Spectroswiss). Data were processed in cumulative time segments, with longer segments yielding incremental increases in (b) resolution and (c) S/N for individual ions. Dots represent experimental HF-OT values using 1 s cumulative segments. Expected theoretical values are shown by the solid (HF-OT, extrapolated from 1 to 4 s values) and dotted (S-OT, HF-OT reduced by 1.7-fold or 1.4 fold factor) lines. The upper pair of lines corresponds to 4+ insulin and the lower pair to 16+ BSA. (d) Distribution plot of detected mass resolutions for multiple individual insulin ions at increasing transient times on the HF-OT analyzer. The dashed lines indicate the top 25% resolution for each transient length. (e) Extracted charge-state distributions after data intensity and resolution filtering at 8-s transients for individual 4+ and 3+ insulin ions and 16+ and 15+ BSA ions on the HF-OT analyzer.

A noise threshold for each segment was calculated as the second maximum of the second derivative of the intensity histogram for 10 scans (Figure S3).^22^ Peaks were detected if their intensity exceeds two (for singly charged peptides) or three times the segment-specific noise level. Subsequently, putative peaks were modeled by fitting a Kernel Density Estimation (KDE) to the local intensity distribution within the corresponding *m*/*z* bin, followed by a nonskewed Gaussian to the KDE output. Peaks exhibiting asymmetric edge intensities or poor fit quality were excluded. The remaining peaks were identified as Gaussian maxima, from which the full width at half maximum (fwhm) mass resolution was calculated. No correction of individual-ion signals using the ‘frequency chasing’ approach was performed.^27^ Instead, nondrifting ions were selected through a combination of intensity and resolution filtering (Figures S4–S5). Initially, the lowest 15% of ions by intensity were discarded to facilitate K-means clustering of the charge states. Clusters for each charge state were then delineated using elliptical contours without any symmetry, intensity, or *m*/*z* range constraint. For the subsequent segments, the ellipse from the 1-s transient was used as a mask to guide intensity filtering. From the resulting points, the top 25% of ions with the highest resolution were retained. These selected ions were then intensity-to-charge calibrated and utilized to obtain the mass and charge distributions. For the 1-s and 8-s transient lengths, the corresponding calibration curves and their slopes (with zero intercept) were used as calibration coefficients (Figure S6).

## Results

### Performance Metrics of the High-Field Orbitrap Analyzer for Individual-Ion CDMS

The Exploris 480 mass spectrometer, equipped with a HF-OT analyzer, has since its introduction in 2020 been widely adopted for high-throughput analysis, primarily of peptides and metabolites.^36^ With the Biopharma option it has also been utilized, to a lesser extent, for the analysis of intact proteins and protein complexes. For the high mass applications, specific instruments, such as the EMR and UHMR mass spectrometers, equipped with a S-OT mass analyzer, were initially developed and are still more commonly used for native MS.^37^ Here we set out to explore whether the Exploris 480 mass spectrometer, which utilizes a HF-OT mass analyzer, could also be used for native MS and in particular Orbitrap-based CDMS. In a first set of experiments we initially aimed at demonstrating that individual ions of intact proteins could be measured by the HF-OT mass analyzer.^35^ We chose bovine serum albumin (BSA ∼ 66.5 kDa) as a proof-of-concept analyte protein. After thorough optimization of the MS parameters, we successfully reached the individual-ion regime, where the intensities of the ion signals appeared quantized. We recorded ∼16,000 individual ions across ∼400 scans using 1-s transients. To benefit from the enhanced CDMS performance, we then extended the recorded transient length to 20 s, which was the maximum duration supported by the hardware configuration. The transients were subsequently processed as cumulative 1-s segments ranging from 0–1 s to 0–20 s to monitor the increase in mass resolution and S/N over transient recording time (Figure 1a). Results are primarily presented at 1 s (prior to extended transient effects), 8 s (an intermediate point with sufficient ion statistics), and 20 s (the maximum transient duration). Next, we inspected the recorded signals of individual ions from BSA (*z* = 16+ at 4236.89 *m*/*z*) and insulin (5.8 kDa, *z* = 4+ at 1443.64 *m*/*z*) to exemplify the performance of the HF-OT analyzer. Over a 20 s transient, the mass resolution increased linearly with transient length, reaching a new record for mass resolution of ∼1,556,000 for BSA and ∼2,690,000 for insulin (Figure 1b). These values are substantially higher than those obtainable with the S-OT (∼949,000 and ∼1,820,000 at 20 s, respectively) and were consistent with theoretical predictions extrapolated from the measurements at shorter transient lengths of 1–4 s. Of note, these mass resolutions are determined in magnitude mode (mFT). In absorption mode (aFT), the achievable resolution did as expected roughly double, reaching over ∼3,560,000 for BSA and ∼5,384,000 for insulin (Figure S2a, b **)**.^28,38^ When examining not only these two stable individual ions, but also the full ion populations, a broader distribution of resolution values was observed due to the inherent variability in stability of each recorded individual ion.^28–32^ After 20 s, the median resolution was ∼614,600 for BSA (Interquartile range, IQR = 142,400 across ∼400 scans) and ∼1,376,900 for insulin (IQR ∼ 278,300 across ∼300 scans), both lower than the theoretical values for stable ions through the whole transient (Figure S2c and Figure 1d). Increased total ion features (8.4k features at 1 s, 55.5k features at 8 s, 54.1k features at 20 s) at long transient durations are consistent with peaks arising from *m*/*z* drift or jumps and background peaks. Those additional features become detectable once the noise threshold decreases following an inverse square root trend. Overall, these observations demonstrate that the HF-OT analyzer enables very high resolution for individual ions and, once stable ions are identified, continues to perform strongly across the full data set.

For the previously selected individual ions, the S/N also increased substantially during the 20 s transient, by ∼2.2-fold for BSA 16+ ions and ∼3.0-fold for insulin 4+ ions when compared to the 1 s acquisition for each individual charge (Figure 1c, noise levels in Table S2). These values followed the hypothesized HF-OT scaling and represent an approximate 1.4-fold improvement over the S-OT (S/N theoretical improvement 1 to 20 s: ∼1.6-fold for BSA and ∼2.2-fold for insulin). The enhanced S/N in HF-OT CDMS consequently improved the charge state assignment (Figure 1e), which can be best appreciated when selecting the most stable ions (top 25% based on mass resolution). Charge with a standard deviation (σ) distribution widths of σ ± ∼0.5e are obtained for stable BSA ions (*z* = 15–16+, across ∼400 scans) and σ ± ∼0.3e for stable insulin ions (*z* = 3–4+, across ∼300 scans) were obtained at 8 s. Charge determination for stable insulin ions approached the goal of 0.2 e, reached by the ELIT CDMS platforms, at which the distribution of charge states becomes sufficiently narrow to allow reliable quantization and exclusion of intermediate charges.^39^ At this charge resolution, mass accuracy is constrained primarily by *m*/*z* uncertainty rather than charge state uncertainty. The difference in charge state uncertainty again reflects distinct gas-phase stability: during a 20 s transient, the insulin ions traveled ∼425 km (experiencing ∼1 collision in the ultrahigh vacuum of the Orbitrap analyzer assuming an ion diameter of ∼3.0 nm) and ions of BSA ∼ 248 km (∼6 collisions assuming an ion diameter of ∼8.0 nm), with the cumulative collisions of the BSA ions substantially affecting their trajectories and limiting the achievable charge accuracy.^27^ By applying stricter resolution filtering, even higher accuracy can be attained, though this reduces the number of ions available for analysis (BSA *z* = 16+: top 50% resolution, ∼2000 ions, ∼0.68e; top 10%, ∼400 ions, ∼0.47e). Together, these observations motivated us to invest further into the data processing workflow, especially for these lower-stability analytes, to exploit the high-resolution and sensitivity of HF-OT analyzer.

### Tailored Data Processing for Stable, Low-Charge Individual Ions

Theoretically ions oscillating in a perfect vacuum within an ideal Orbitrap mass analyzer experience indefinitely stable trajectories, allowing them to be trapped “forever” and allowing extremely precise measurement of their oscillation frequency and, consequently, their mass. In practice, the temporal evolution of an individual ion signal depends on collisions with the background gas present in the ultrahigh vacuum of the Orbitrap analyzer and the ion’s response to these interactions. The longer the transient recording, the higher the likelihood that such events occur and the ion’s trajectory will destabilize. Several groups have developed advanced processing tools to correct for shifts in ion frequencies, to take full advantage of CDMS measurements.^22,23,26^ Our previous studies have focused primarily on high-mass ions, which most often experience small frequency drifts related to gradual removal of residual solvent molecules.^16^ We demonstrated that such frequency drifts can be effectively tracked and corrected using short-time Fourier transform analysis (frequency chasing algorithm,^27,28^ Figure S1, Materials and Methods).

Orbitrap-based CDMS of smaller analytes (<150 kDa) present additional challenges as described in the recent ECCR-CDMS study.^32^ The behavior of such ions is even more unpredictable, as their frequency jumps are sudden and larger, making them harder to track than the gradual shifts caused by desolvation of highly charged ions. The longer the transient recording times, the longer are the individual ion trajectories. For instance, during a 20 s period, insulin travels an impressive 425 km in the HF-OT, while BSA travels 248 km. These long ion trajectories result in more collisions with gas background molecules, increasing the likelihood of frequency jumps. In addition, as ion charge approaches a single charge, the real ion signals remain masked within the noise level, especially during the first seconds of the transients, preventing the separation of true ion signals from noise.

To address these challenges, we had to implement a data processing approach tailored for low-*m*/*z* ions that introduces stricter peak-picking criteria and refined resolution calculations (Figure 2, Materials and Methods). We chose BSA to benchmark our approach because we already observed that these ions poorly survive over a full 20-s transient.^32^ First, we determined the experimental internal noise threshold for each cumulative time-step transient length (Figure S3, Table S2). Only the peaks with an S/N greater than 3 (adjusted to 2 for singly charged ions) were considered for further data processing (Figure 2a). Estimating the noise band diminishes false positives especially for noise-limited signals. We then fitted the retained peaks to Gaussian distributions, excluding those with asymmetric shapes or poor fits (Figure 2b). For the remaining peaks, we computed their fwhm mass resolution (Figure 2c). Despite this preliminary peak selection, many ions still displayed frequency drifts with attenuated intensities and broad fwhm mass resolution distribution, indicating that additional processing was required to filter out remaining unstable ions (Figure 2d). We consequently applied intensity and top-25% resolution filtering (Figure 2e, Figures S4–S5). The 2D histograms revealed smaller clusters of points where the standard deviation on the charge decreases with transient length, highlighting the benefits of longer transients. Finally, we performed intensity-to-charge calibration (Figure S6), and generated mass histograms (Figure 2f).

**Figure 2:**
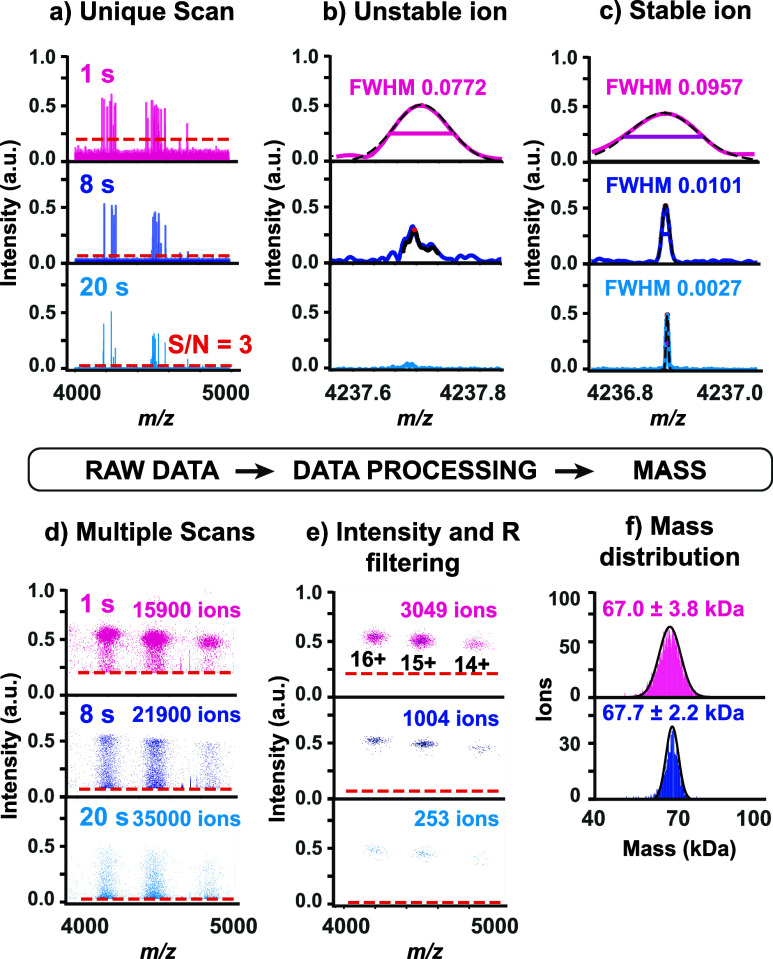
Intensity- and resolution-based filtering improves individual-ion CDMS on the HF-OT. (a) Mass spectra of individual BSA ions in cumulative transients of 1, 8, and 20 s. Gaussian fitting (b) for nonideal unstable signals that are successively excluded and (c) for ideal stable ions from which the fwhm and resolution are calculated as depicted. (d) 2D histograms of ion signals across multiple scans, after removal of non-Gaussian peaks. The intensity decreased owing to substantial ion drift. (e) 2D histograms after intensity and top-25% resolution filtering, with larger bins for clarity. (f) Mass histograms generated following intensity-to-charge calibration for 1-s and 8-s transients. The histogram at 20-s transient is not depicted due to insufficient features. In all subfigures the internal noise threshold is indicated by red dashed lines.

The resulting experimental mass distributions were accurate and fully consistent with the expected mass at both 1 and 8 s. This confirms that the data processing pipeline performs reliably, efficiently removing unusual mass distortions such as skewed or satellite peaks. Our data processing retains only stable ions, while unstable ones are effectively filtered out. This approach confidently resolves low-charge, near-noise signals, accurately determines peak shapes, minimizes artifacts, and ensures mass resolution reflects true analyte properties. Most importantly, it will allow proper CDMS analysis of low masses that were so far undetectable using Orbitrap-based CDMS. The theoretical predictions of the S/N led us to hypothesize that singly charged ions could now be detected using the HF-OT analyzer (Figure 3a), but not on the S-OT analyzer.

**Figure 3:**
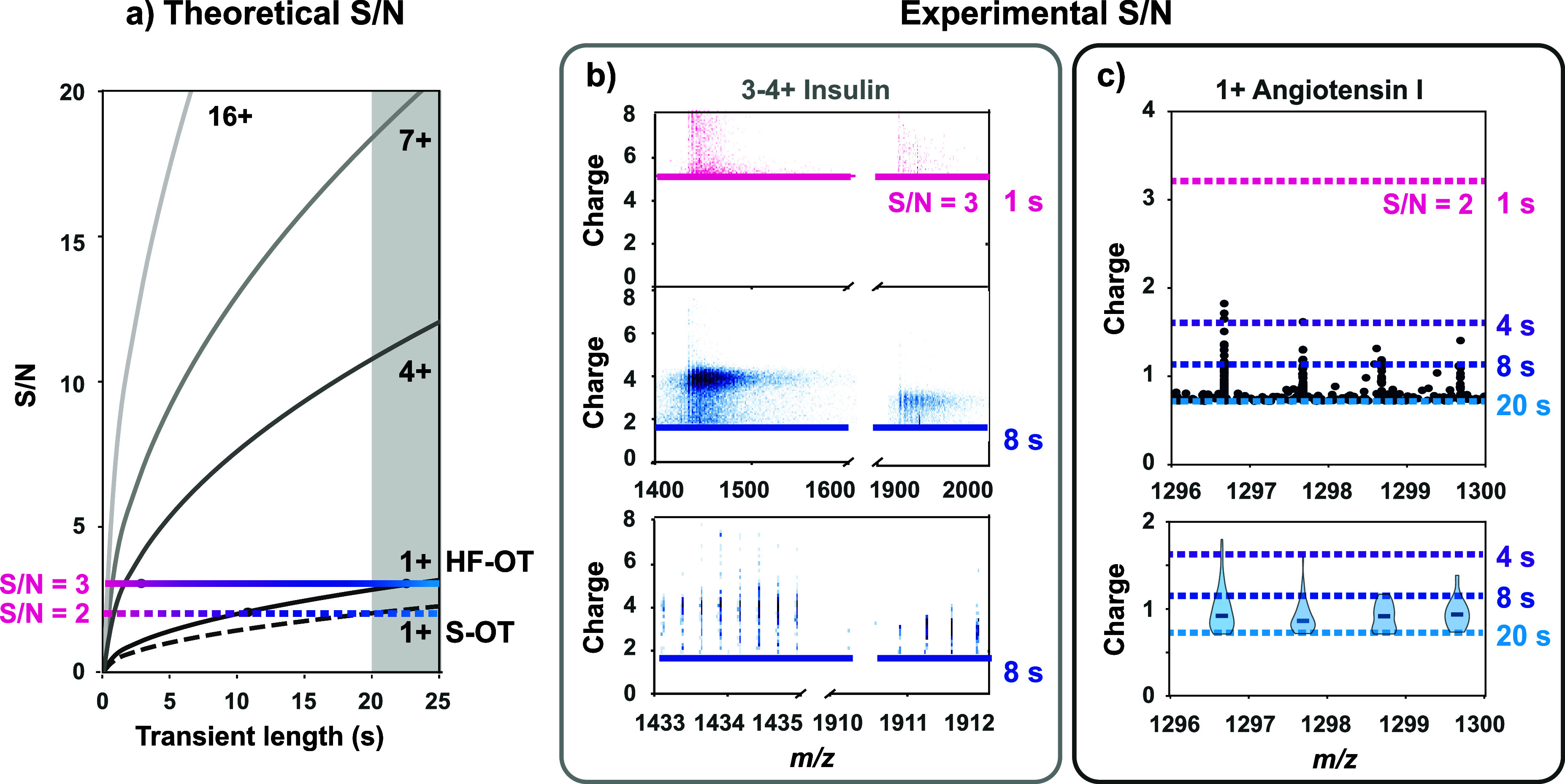
Robust detection of individual, singly charged ions by Orbitrap-based CDMS. (a) Theoretical obtainable S/N values on the HF-OT (solid) and S-OT (dashed) geometries over a 25-s transient, depicted for ions of charge states *z* = 1, 4, 7+, and 16+. Gray shading indicates the current HF-OT hardware constraint for maximum transient length (∼20 s). (b) Detection of individual triply and quadruply charged ions of insulin. Raw 2D histograms of *m/z versus* charge are shown for ions of insulin at 1 s (∼8000 ions, S/*N* > 3) and 8 s (∼55,500 ions, S/*N* > 3). Zoomed view of 8-s histogram highlights 4+ and 3+ charge states with resolved isotopic distributions. (c) Detection of individual singly charged ions of angiotensin I. Scatter and violin plots of *m/z versus* charge demonstrating the robust detection of singly charged angiotensin I ions at 20-s transients (S/*N* > 2). Dashed lines indicate the S/*N* = 2 thresholds at different transient lengths. In this latter plot, the total number of ion events reaching above the noise threshold is 0, 3, 74, and 146 at transient lengths of 1, 4, 8, and 20 s, respectively. At 20 s, 91% of these events are observed in the expected bins, corresponding to the isotope peaks of angiotensin I. Notably, at 1297 *m*/*z*, the observed resolution for the singly charged angiotensin I ion was 3.4 million in magnitude mode. Similar results were obtained for the peptide bradykinin as shown and described in Figure S7.

### Singly Charged Individual Molecule Ion Detection

Detecting individual ions carrying a single charge in Orbitrap-based CDMS has long been regarded as impossible, as the maximal acquisitions accessible on commercial instruments leave the signal of these ions largely buried below the noise level. For instance, a perfectly stable 1+ ion would require a transient recording of at least ∼42 s to reach a signal above the noise level (S/N = 3, ∼21 s for S/N = 2) in a S-OT analyzer (Figure 3a). HF-OT could reach the same S/N with a 23-s transient. However, this exceeds the current ∼20-s stability limit in the maximum transient duration. A S/N of 2 can already be obtained in just 10 s, making it theoretically feasible to distinguish 1+ ion signals while exploiting the maximum transient length of 20 s.

To investigate near noise level signals, we first analyzed the time dependence of noise using ions of insulin as a model system. At 1 s, charge distributions remained unresolved, and the charge was mostly misassigned, whereas at 8 s, both the 4+ and 3+ patterns matched with theoretical simulations, illustrating the benefit of combining longer transients for HF-OT CDMS (Figure 3b). Building on this insight, we analyzed a mixture of two commonly used model peptides: angiotensin I and bradykinin (Figure 3c and Figure S7). For the first time, making use of the HF-OT analyzer and long transient recording, individual ions of singly charged peptides could be successfully detected by Orbitrap-based CDMS. In adjacent regions in the *m*/*z* window, the noise-only regions remained largely devoid of alike counts. Both peptides form a clearly resolved isotopic pattern with individual ion signals above the noise threshold (S/N = 2) after 20 s. For this detection, extending the transients beyond 10 s proved to be crucial, as it clearly increased ion counts and improved the charge assignment from ∼1.5 at 10 s to ∼1.0 at 20 s (Figure S7). These results demonstrate that with long transient HF-OT CDMS individual ions of every nature can be detected and mass analyzed, from very high mass to very low mass, and from very highly charged (hundreds of charges) to minimally charged, i.e., 1+, whereby the long transients improve the S/N, the charge assignment and thus also the mass accuracy.

### High-Field Orbitrap CDMS Proves Versatility over an Extended *m*/*z* Range from 1 to 500 kDa

Above we demonstrated the benefits of the HF-OT for CDMS on small proteins and even singly charged peptides, setting a new lower mass and charge limit for Orbitrap-based CDMS. Notably, most native and CDMS applications are focused on high-mass analytes and analyzed using predominantly the UHMR mass spectrometer,^1,2^ although it has been demonstrated that also on the Exploris 480 mass spectrometer intact antibodies can be mass analyzed by using ensemble native MS.^40^ Next, to further explore the potential of the HF-OT for native MS of macromolecules, we first measured a monoclonal antibody (i.e., trastuzumab, mAb, ∼150 kDa). The individual mAb ions analysis using short-time Fourier transform and frequency-chasing correction yielded a mass of 148 kDa (±∼2 kDa) with a charge accuracy of σ ∼ ±0.3e at 20 s (Figure 4a; low-*m*/*z* processing at 8 s, Figure S8). These high-accuracy values were obtained by following and correcting ion frequencies exclusively for desolvated, nonfragmented ions through (i) removal of duplicate, incomplete, and intersecting traces (Figure S1), and (ii) correction via internal calibration of slight temporal intensity drift across charge states due to background gas friction (Figure S9).^16^ Overall, the HF-OT CDMS demonstrates better charge accuracy for individual mAb ions, exceeding previously reported values on the S-OT (±∼0.6–0.8e at 25 s).^32^

**Figure 4:**
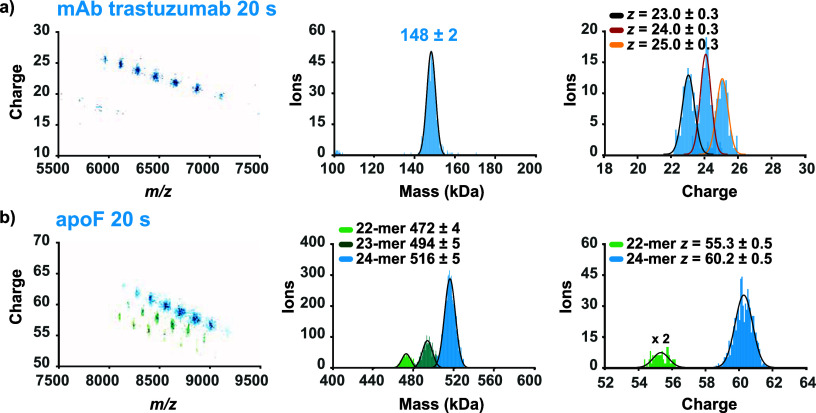
Applications of HF-OT individual-ion CDMS for high *m/z* species. (a) 20-s 2D histogram of individual IgG-mAb ions and their mass distribution. Charge distributions of individual mAb ions (*z* = 23–25+) are clearly resolved. (b) 20-s 2D histogram and corresponding mass histogram of individual apoF ions, revealing distinct mass distributions for the 22-mer, 23-mer, and 24-mer species. Charge-state distributions for ∼100 55+ ions (×2) from the 22-mer and ∼700 60+ ions from the 24-mer are shown with fitted Gaussian curves.

We next investigated apoferritin (apoF, ∼500 kDa), an even larger macromolecular analyte. As previously reported, recombinantly produced apoferritin is an interesting candidate to assess CDMS performance.^28^ Recombinant apoferritin consists of distinct multimeric assemblies, namely 22, 23 and 24-mers, and therefore following native electrospray it produces also two specific isobaric ion species: the 22-mer 55+ and 24-mer 60+, both at *m*/*z* ∼ 8556.^28^ We first attempted to detect apoF in native ensemble mode and were able to obtain a spectrum after meticulous tuning of all transmission lens DC potentials (Table S1). Once the transmission was optimized in ensemble native MS, we turned to CDMS. The Orbitrap Exploris 480 mass spectrometer achieved performance metrics that matched those we obtained earlier when using the UHMR mass spectrometer,^28^ showing robust oligomer separation, with a standard deviation of mass at only 5 kDa, and a charge state standard deviation of just ±∼0.5e (Figure 4b). Further improvements in charge accuracy could be obtained through enhanced desolvation via gas handling, potentially extending applicability of CDMS to analytes in the MDa range (Figure S10).

## Conclusions and Discussion

Here, we explored the use of the HF-OT analyzer for CDMS analyses across a broad *m*/*z* range, demonstrating its advantages over the S-OT analyzer. The HF-OT analyzer achieved a 1.7-fold higher resolution and a 1.4-fold higher S/N, confirming its predicted boosted performance as compared to the S-OT analyzer (Figure 1). The calculated charge accuracy approached ±∼0.3e (Figure 1) at 8 s, bringing Orbitrap-based CDMS closer to the almost perfect charge assignment reported for state-of-the-art ELIT-CDMS platforms of ±∼0.2e at 1.5 s, or in just 600–700 ms using cryogenically cooled electronics under highly optimized conditions.^28,39,41^ Such improvements, almost a 2-fold gain relative to the S-OT analyzer, require both ion stability during the transient and complete desolvation. BSA and apoF represent those specific exceptions: BSA likely lacks sufficient stability during the transient, while apoF remains partially solvated prior to detection. As a result, charge accuracies remain around ±∼0.5e at 8 s for BSA and at 20 s for apoF. In addition, improvement in charge accuracy enables achieving similar charge resolution with approximately half the transient length, effectively doubling the duty cycle for comparable charge resolution. This reduction in acquisition time is a key advantage for adapting CDMS to size exclusion chromatography, capillary electrophoresis and other LC-based workflows used to introduce the samples.^19,42^

Nevertheless, these improvements enabled for the first time the analysis of singly charged peptides, including angiotensin I and bradykinin (∼1 kDa, Figure 3). Previously, on a S-OT low charged ions of ∼12 kDa (e.g., cytoC *z* = 2+) could be measured, but signals from singly charged ions were obscured by noise, even at maximum acquisition times.^32,43,44^ The successful detection of singly charged peptide ions establishes a fundamentally new lower limit for mass and charge, significantly expanding the analytical range, resolution, and sensitivity of Orbitrap-based CDMS. An interesting future application of this new capability could be top-down analysis, whereby the HF-OT CDMS would allow access to singly charged, low-abundance fragment ions, species that could remain undetectable by standard Orbitrap ensemble measurements.^45^

At high *m*/*z*, the HF-OT analyzer enabled accurate characterization of large macromolecular assemblies, such as apoferritin (∼500 kDa, Figure 4), performing robustly without hardware modifications beyond those needed to record long transients. ApoF is just a single example of where HF-OT CDMS could excel. Additionally, measuring analytes that fall within the mass range of conventional native MS but remain elusive due to their proteoform complexity such as highly heterogeneous glycoproteins, would benefit from the advances described here. With further hardware modifications, including elevated HCD gas pressures for improved collisional cooling and upgraded electronics to enhance ion transmission and detection, the Orbitrap Exploris 480 mass spectrometer could further extend the usable mass range into the megadalton scale where heterogeneity is even more prominent, comparable to the Orbitrap UHMR platform mass range.

In conclusion, the HF-OT analyzer, as described here, provides a next-generation platform that substantially expands the analytical capabilities of CDMS, with gains in resolution, sensitivity, charge- and mass accuracy. It will benefit the analysis of low- and high-mass analytes, including highly mass-heterogeneous samples such as glycoproteins and biopharmaceutical products that continuously are expanding in molecular complexity.

## Supplementary Material



## Notes

MS parameters used for HF-OT CDMS measurements on the Orbitrap Exploris 480 mass spectrometer; measured HF-OT noise band for cumulative time segments over 10 scans; frequency chasing for low *m*/*z* ions; mFT and aFT fwhm mass resolution; noise definition; intensity- and resolution-based filtering for BSA ions (16 < *z* < 14); intensity- and resolution-based filtering for mAb ions (20 < *z* < 26); intensity-to-charge calibration for CDMS measurements; detecting individual singly charged peptide ions; mAb mass and charge resolution with low *m*/*z* data processing workflow; histograms representing intensity drift and average charge per time segment as a function of transient length; ApoF neutral losses (PDF)
