# Supplementary material for: Analyses of Individual Singly Charged Ions Using a High-Field Orbitrap Analyzer
Source: Anal Chem. 2026 May 12;98(20):15250–8. doi: 10.1021/acs.analchem.6c02125 (PMC13218364; doi:10.1021/acs.analchem.6c02125)
Supplement: Supplementary file 1 [file ac6c02125_si_001.pdf]

Supporting Information for:

Analyses of Individual Singly Charged Ions using a High-Field Orbitrap Analyzer

Elena Giaretta[1], Evolène Deslignière[1,2], Eduard H. T. M. Ebberink[1], Tobias P. Wörner[3], Arjan Barendregt[1], Kyle L. Fort[1,3], Alexander A. Makarov[1,3], Albert J. R. Heck[1]*

---

[1] Biomolecular Mass Spectrometry and Proteomics, Bijvoet Center for Biomolecular Research and Utrecht Institute for Pharmaceutical Sciences, Utrecht University, Utrecht 3584 CH, the Netherlands

[2] Université Paris-Saclay, CNRS, Institut de Chimie des Substances Naturelles, UPR 2301, 91198 Gif-sur-Yvette, France

[3] Thermo Fisher Scientific GmbH, Bremen 28199, Germany

*Correspondence to: a.j.r.heck@uu.nl

Table of contents:

Table S1. MS parameters used for HF-OT CDMS measurements on the Orbitrap Exploris 480 mass spectrometer. ..S2

Table S2. Measured HF-OT noise band for cumulative time segments over 10 scans. S3

Figure S1. Frequency chasing for low *m/z* ions. S4

Figure S2. FWHM Mass Resolution. S5

Figure S3. Noise definition. S6

Figure S4. Intensity- and resolution-based filtering for BSA ions ($16<z<14$). S7

Figure S5. Intensity- and resolution-based filtering for mAb ions ($20<z<26$). S8

Figure S6. Intensity-to-charge calibration for CDMS measurements. S9

Figure S7. Detecting individual singly-charged peptide ions. S10

Figure S8. mAb mass and charge resolution with low *m/z* data processing workflow. S11

Figure S9. Intensity drift. S11

Figure S10. ApoF neutral losses. S12

References S12

Table S1. MS parameters used for HF-OT CDMS measurements on the Orbitrap Exploris 480 mass spectrometer.

*To favor the detection of apoF over lower *m/z* ions, the following instrumental parameters were adjusted: the Inter Flatapole Lens was set to 8 V, the Bent Flatapole DC to 8 V, and the Bent Flatapole Gradient to 50 V.

** From Protein Data Bank (mAb 4HJG, BSA 4F5S, Insulin 1A7F)

MS parameters	apoF*	mAb	BSA	CytoC	Insulin	1+
m/z range	6500-12000	5000-8000	4000-5000	1000-3000	1350-2000	600-2000
Spray voltage (kV)	1.4	1.3	1.2	1.2	1.3	1.3
Isolation range	8000-11000	5000-8000	4000-5000	1000-3000	1350-2000	600-2000
Booster sampling frequency (Hz)	651042	651042	651042	976562	976562	976562
Transient length	20	20	20	20	20	20
# Buffer scan	1	3	3	3	3	3
Acquisition time (h)	1.5	2	3	2	2	2
AGC mode	fixed	fixed	fixed	fixed	fixed	fixed
Ion injection time (ms)	6	10	500	1	10	2
Microscans	1	1	1	1	1	1
S-lens RF	200	200	200	200	50	50
Noise threshold	0	0	0	0	0	0
Trapping gas pressure	1.5	0.8	0.1	0.1	0.1	0.1
UHV readout (mbar)	6.57e-12	3.12e-11	1.83e-11	1.93e-11	1.53e-11	1.78e-11
Collision gas	N_2	N_2	N_2	N_2	N_2	N_2
In-source CID fragmentation	90	0	0	0	0	0
HCD voltage (V)	10	10	10	0	0	0
Diameter (nm)**	-	9.2	8.0	-	3.0	-

Table S2. Measured HF-OT noise band for cumulative time segments over 10 scans. Transient data for a reported *m/z* range were zero-filled one or two times and apodized selectively, with some spectra processed using a Hamming apodization window. Data analysis of apoF follows a separate code provided elsewhere, using prominence-based peak picking.[1]

	**mAb**	**BSA**	**CytoC**	**Insulin**	**1+**
m/z range	5000-8000	4000-5000	1600-2500	1350-2000	1000-2000
**Noise fitting curve Hamming apodization / y=a*x^(-b)**					
a	7.86e-2	7.25e-2	6.05e-2	5.90e-2	5.62e-2
b	0.51	0.50	0.50	0.50	0.50
R^2	0.9994	0.9998	0.9999	1.000	1.000
**Noise fitting curve RAW / y=a*x^(-b)**					
a	1.26e-1	1.17e-1	9.72e-2	9.42e-2	9.11e-2
b	0.51	0.51	0.50	0.50	0.50
R^2	0.9996	0.9998	0.9999	0.9999	1.000

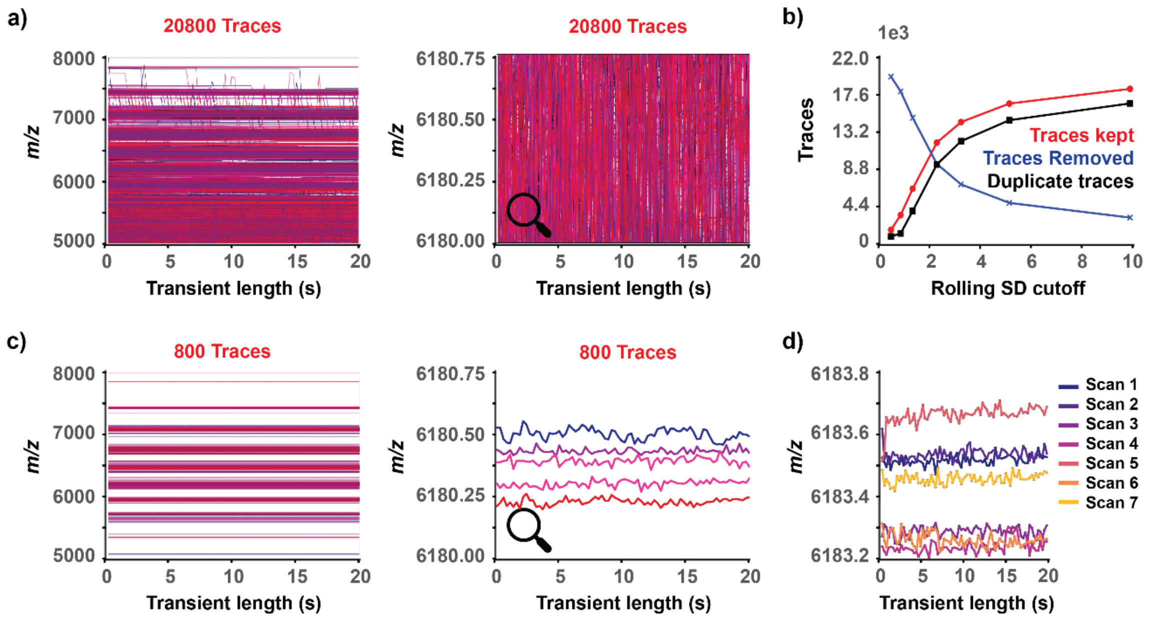

Figure S1. Frequency chasing for low *m/z* ions.[1] **(a)** Individual traces of mAb ions within 5000-8000 *m/z* window (*z*=20-26+) obtained via the 'frequency chasing' approach, without any filtering. The zoomed-in graph in the 6180-6200 *m/z* region shows some vertical segments related to wrongly traced ions jumping up and down to different frequencies. **(b)** Filtering of correctly traced ions using a rolling standard deviation cutoff between four consecutives segments of 0.256 s.[2] As the standard deviation threshold increases, the filtering becomes less strict, allowing more traces to be retained. Some of these traces may be duplicates, where incomplete sets select the nearest trace to reach the transient end. **(c)** Filtering of correctly traced ions using a $\sigma < 0.1$ and complete trace sets. The zoomed-in graph in the 6180 - 6200 *m/z* region shows the absence of vertical jumps. **(d)** Zoomed-in graph displays some of the stable ion traces, each shown in a distinct color corresponding to a different scan.

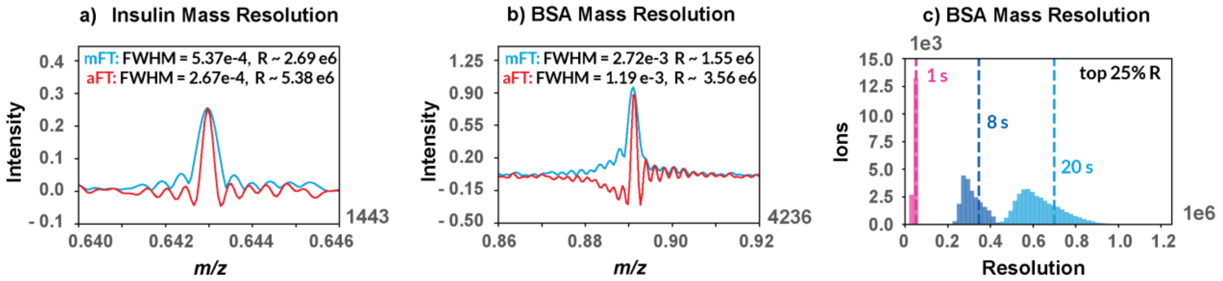

Figure S2. FWHM Mass Resolution. (a,b) Individual-ion (insulin, *z*=4+ and BSA, *z*=16+) signal recorded from **a** 20 s transient, processed with mFT vs. aFT. aFT (red) yields, as expected, a ~2-fold improvement of the FWHM mass resolution over mFT (light blue) and results in a resolution over 5.38 million at 1443.36 *m/z* for insulin and 3.56 million at 4236.89 *m/z* for BSA.[3] **(c)** Histogram depicting the distribution of observed mass resolution for BSA ion at increasing transient recording times. Individual ions (BSA, *z*=14-16+, 1.6k features at 1 s, 21.9k features at 8 s, 35.0k features at 20 s) were collected across ~400 scans. Here, all ions with intensity greater than S/N>3 were used. Drifting "unstable" ions are not filtered out in this figure.

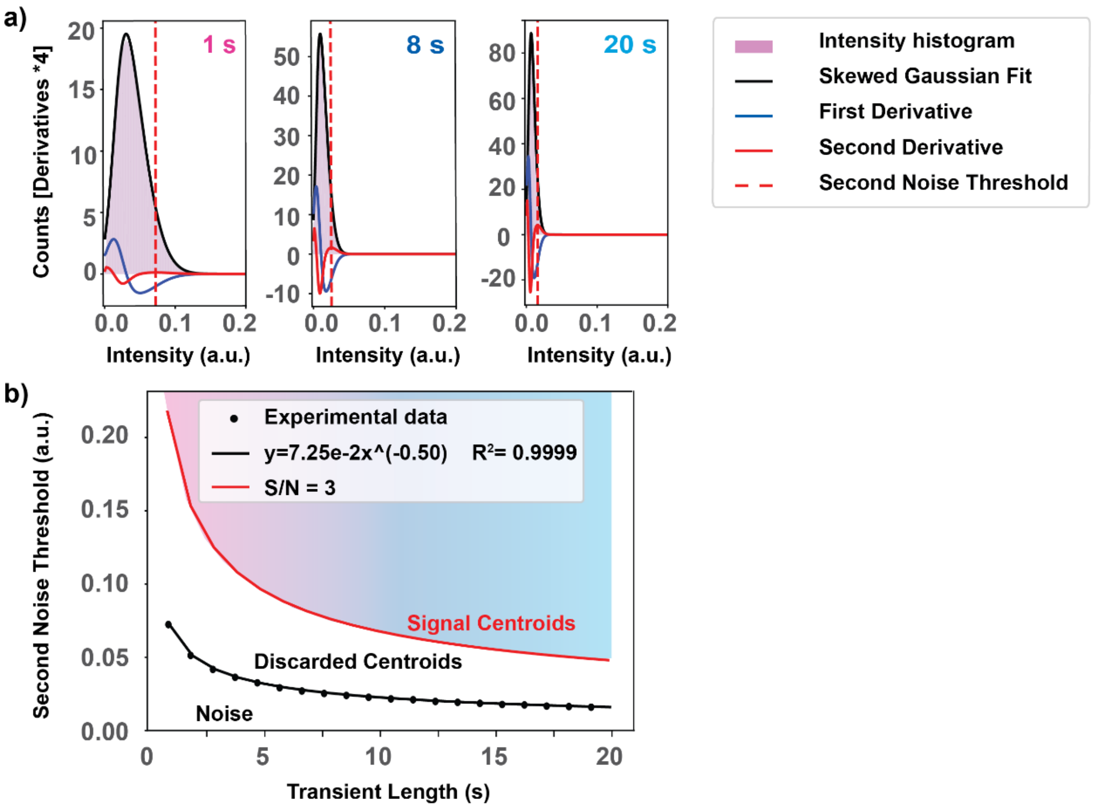

Figure S3. Noise definition.[4] **(a)** Definition of BSA noise levels for 1-s, 8-s, and 20-s transients within 4000-5000 *m/z* window (*z*=14-16+) acquired over 10 scans. The normalized intensity histogram of the signal shown in pink was fitted with a skewed Gaussian function. The first and second derivatives of the fitted curve were computed and are shown in blue and red, respectively. Both derivatives were magnified by a factor of four for improved visualization. The noise level was defined as the second maximum of the second derivative of the fitted function and is indicated as vertical red dotted line. **(b)** Measured BSA segments noise band for cumulative time segments. The black dots represent experimental HF-OT noise data, fitted with an inverse square root function as the solid black line. This function is multiplied by 3 to set the S/N ratio threshold.

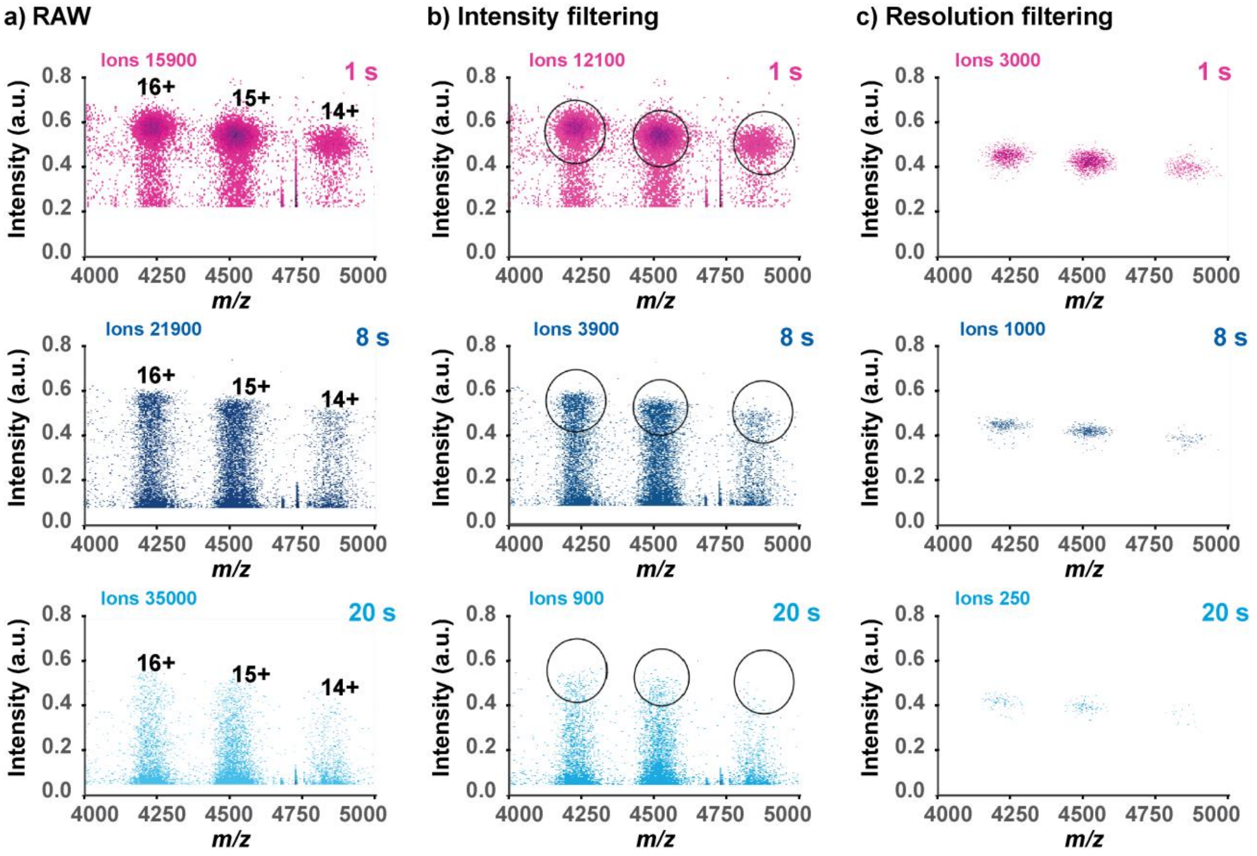

Figure S4. Intensity- and resolution-based filtering for BSA ions (16<z<14). (a) 2D histogram of individual BSA ion signals within 4000-5000 *m/z* window acquired over ~400 scans using 1-s (top), 8-s (middle), and 20-s (bottom) transients after peak picking (**Fig. 2d**). **(b)** K-means clustering identifies charge states, with clusters defined by elliptical contours without any constraints on symmetry, intensity or *m/z* range. The shape and position of the elliptical contours highlight the quality of the charge state cluster, with more symmetrical and compact clusters indicating a higher feature number of stable, non-splitting ions. The ellipses derived from the 1-s transient are intensity masks for the 8-s and 20-s transients. **(c)** Of the initial ions (depicted in **(a)**) only ions with the highest resolution (top 25%) are retained, representing ~18.9%, 4.6% and 0.7 %, when recording transients of 1, 8 and 20 s, respectively (**Fig. 2e**).

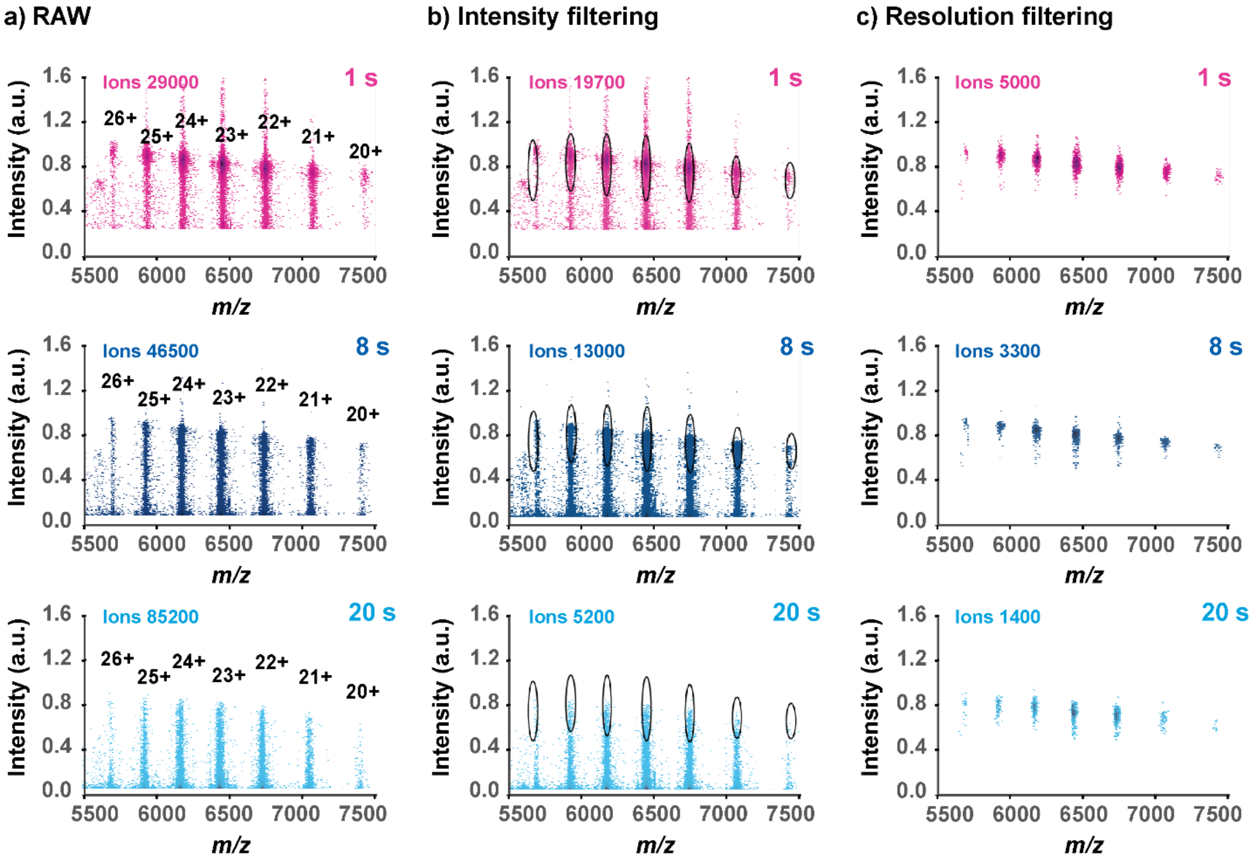

Figure S5. Intensity- and resolution-based filtering for mAb ions (20<z<26). (a) 2D histogram of individual mAb ion signals within 5000-8000 *m/z* window acquired over ~300 scans using 1-s (top), 8-s (middle), 20-s (bottom) transients after peak picking. **(b)** K-means clustering identifies charge states, with clusters defined by elliptical contours without any constraints on symmetry, intensity or *m/z* range. The ellipses derived from the 1-s transient are intensity masks for the 8-s and 20-s transients. The first and last cluster (*z*=26,20) are not included in the calculation of the mass histogram, as they do not follow to the theoretical ion ledge. **(c)** Of the initial ions (depicted in **(a)**) only ions with the highest resolution (top 25%) are retained, representing ~1.7%, 0.7% and 0.2 %, when recording transients of 1, 8 and 20 s, respectively.

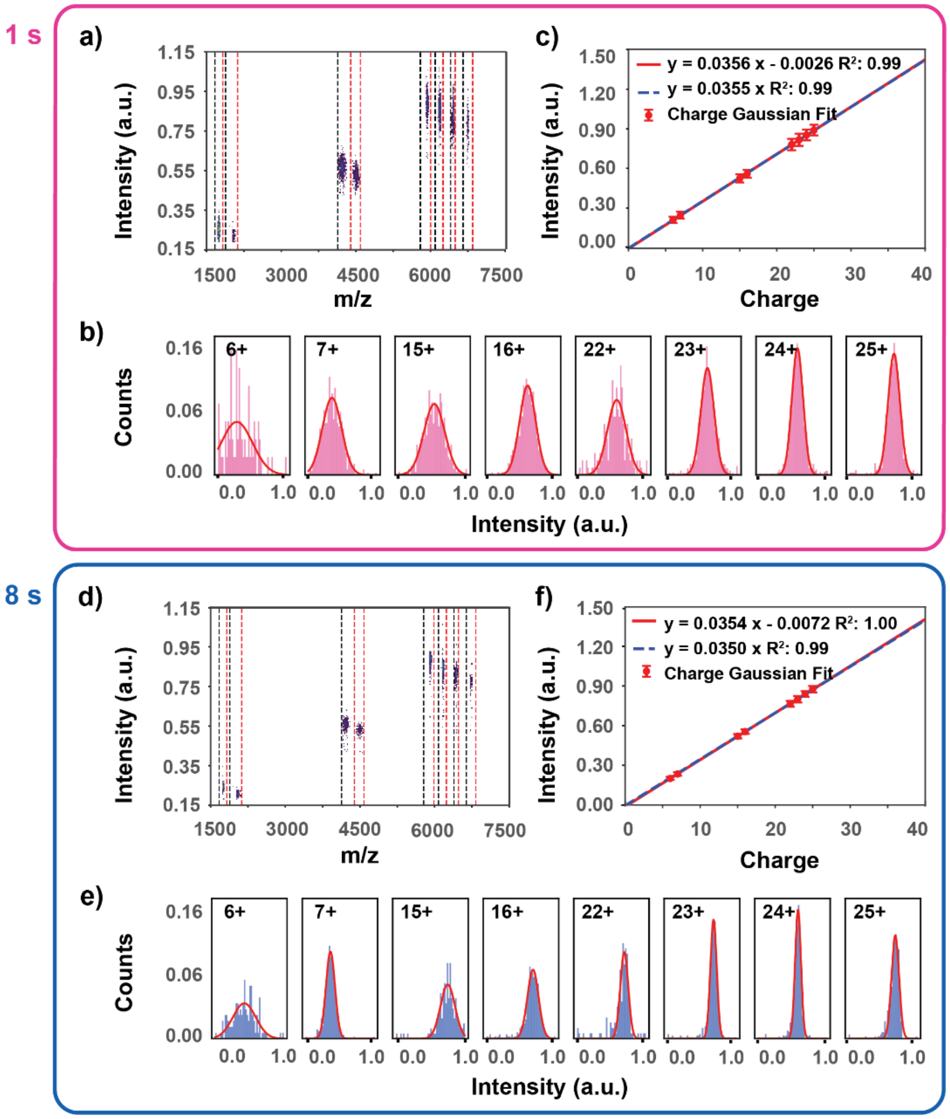

Figure S6. Intensity-to-charge calibration for CDMS measurements.[5] **(a)** 2D histogram of individual cytoC (*z*=6-7+), BSA (*z*=15-16+), and mAb (*z*=22-25+) ion signals at 1 s. *m/z* ranges are sliced and assigned to their corresponding calibrant charge states. **(b)** Gaussian fitting of charge states identified and sliced in panel **a**. The mean intensity (and σ) associated with each given charge state is determined from its fit and used to build the intensity-to-charge calibration curve shown in **(c)**. A linear fit is then performed, and the resulting slope (with zero intercept) serves as calibration coefficient. **(d-f)** The same calibration procedure was applied after 8 s. Note that the slope of the calibration curve decreases slightly due to intensity drift.

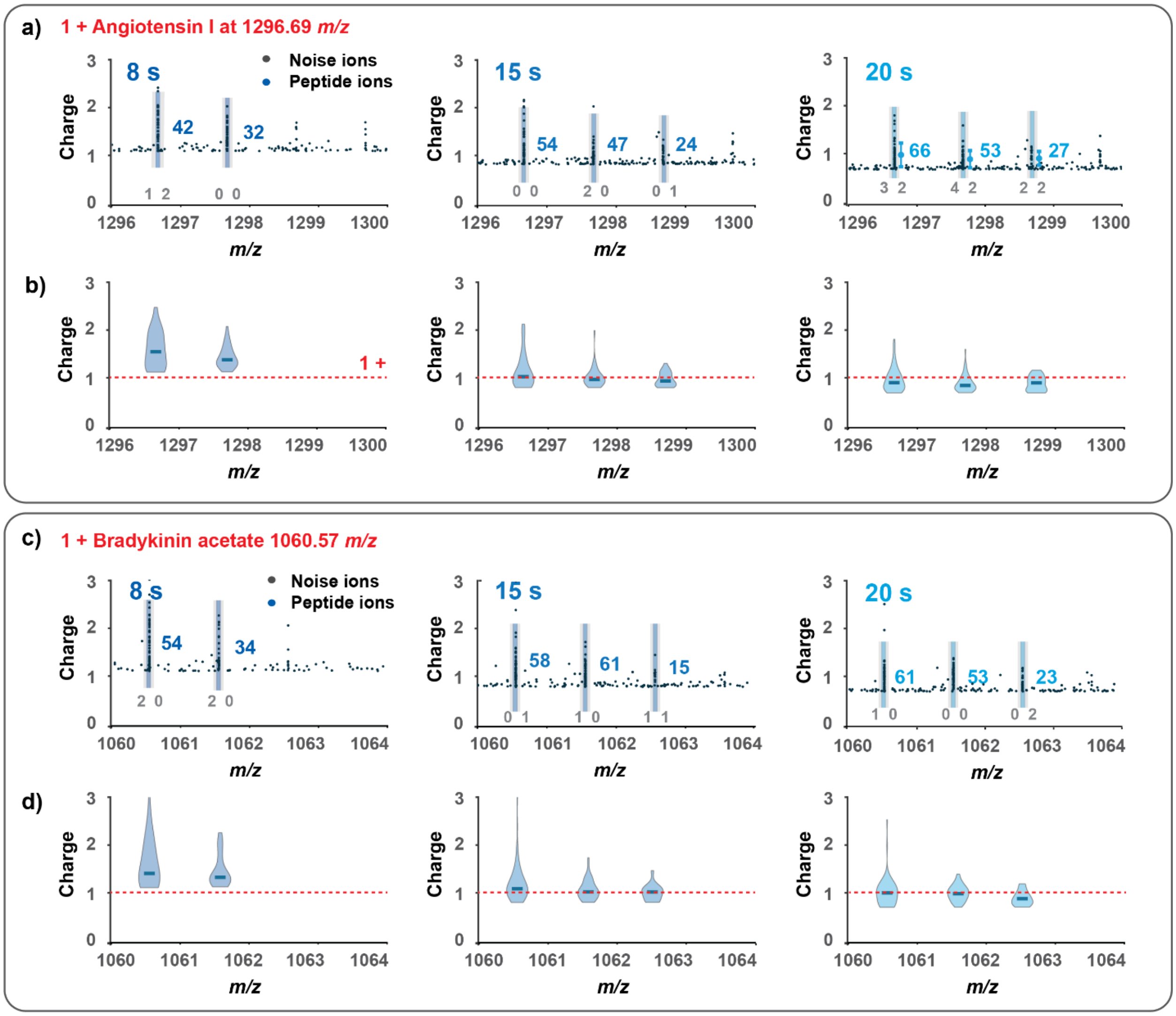

Figure S7. Detecting individual singly-charged peptide ions. Scatter and violin plots *m/z* versus charge illustrating singly-charged of Angiotensin I (1296.69 *m/z*) **(a-b)** and Bradykinin (1060.57 *m/z*) **(c-d)** ions at 8-, 15-, and 20-s transients (~300 scans, S/N >2). Scatter plot blue areas highlight *m/z* bins containing at least 15 ions, and adjacent gray regions neighboring bins and their respective ion counts. Violin plot showing the average values and their deviation from the singly-charged case (dashed red line). At 20 s the obtained resolutions are 3.4 and 4.3 million for Angiotensin I (1296.69 *m/z*) and Bradykinin (1060.57 *m/z*), respectively.

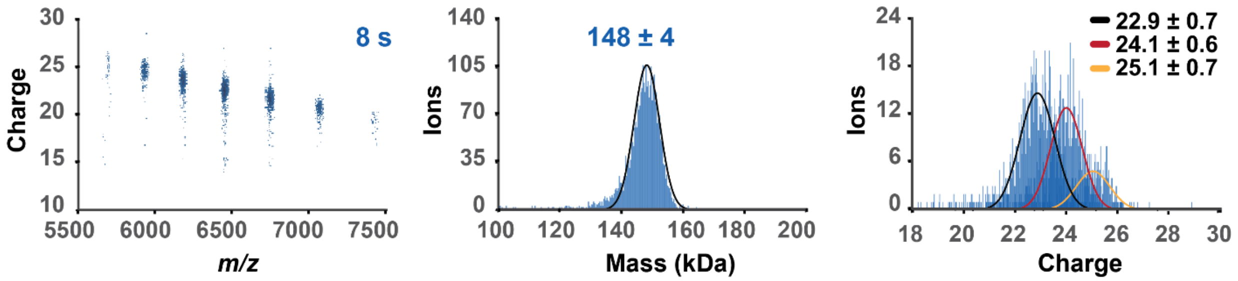

Figure S8. mAb mass and charge resolution with low m/z data processing workflow. 8-s 2D histogram of individual mAb ions after intensity and resolution filtering (top 25%). Mass histogram following 8-s intensity-to-charge calibration. Charge distributions at 8-s transients for individual 23+, 24+, and 25+ mAb ions. In this case, the frequency chasing workflow with correction of ion drifts performs better (**Figure 4**) as the mAb already falls in the high mass range (150 kDa).

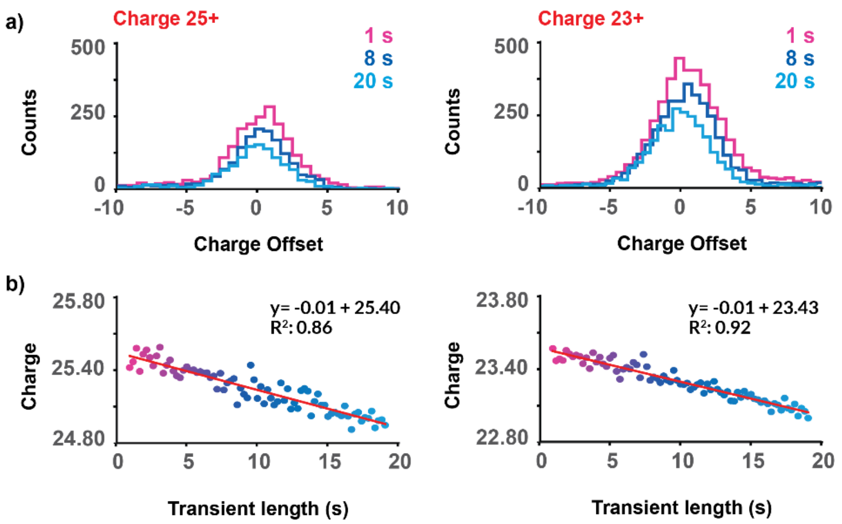

Figure S9. Intensity drift.[2] **(a)** Histogram of charge state offset for segmented time-domain transients (0-1 s, 1-2 s, ..., 19-20 s) measured across ~ 300 scans for individual mAb ions with charge states 25+ and 23+. No tracing of individual-ion signals using the 'frequency chasing' approach was utilized.[1] Intensity-to-charge was computed with the 1-s transient calibration coefficient. The intensity distribution exhibits a progressive leftward shift between early and late time segments. **(b)** Average charge per time segment as a function of transient length and its linear fitting (red solid line).

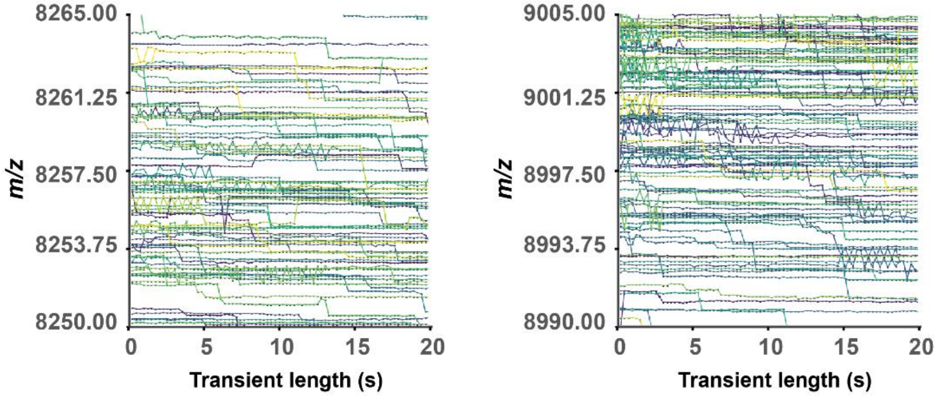

Figure S10. ApoF neutral losses.[3] Example of two zoomed-in *m/z* ranges (62+ and 57+ apoF for the 24-mer), illustrating some of the traces from 260 scans, with each color representing a distinct scan and highlighting gradual frequency drift from multiple neutral solvent losses.

References

1. Wörner TP, Aizikov K, Snijder J, Fort KL, Makarov AA, Heck AJ. Frequency chasing of individual megadalton ions in an Orbitrap analyser improves precision of analysis in single-molecule mass spectrometry. *Nature chemistry* **14**, 515-522 (2022).

2. Ebberink EHTM*, et al.* Prolonged Trapping of Adeno-Associated Virus Capsids Reveals that Genome Packaging Affects Single-Ion Mass Spectrometry Measurements. *Journal of the American Chemical Society* **147**, 10925-10934 (2025).

3. Deslignière E*, et al.* Ultralong transients enhance sensitivity and resolution in Orbitrap-based single-ion mass spectrometry. *Nature Methods* **21**, 619-622 (2024).

4. Kostelic MM*, et al.* UniDecCD: Deconvolution of Charge Detection-Mass Spectrometry Data. *Analytical Chemistry* **93**, 14722-14729 (2021).

5. Wörner TP, Snijder J, Bennett A, Agbandje-McKenna M, Makarov AA, Heck AJR. Resolving heterogeneous macromolecular assemblies by Orbitrap-based single-particle charge detection mass spectrometry. *Nature Methods* **17**, 395-398 (2020).
